# Temporal changes in the prevalence of childhood asthma and allergies in urban and rural areas of Cyprus: results from two cross sectional studies

**DOI:** 10.1186/1471-2458-11-858

**Published:** 2011-11-11

**Authors:** Ourania Kolokotroni, Nicos Middleton, Nicolas Nicolaou, Spyros Pipis, Kostas N Priftis, Donald K Milton, Panayiotis K Yiallouros

**Affiliations:** 1 Cyprus International Institute for Environmental and Public Health in Association with Harvard School of Public Health, Cyprus University of Technology, Limassol, Cyprus; 2 Department of Nursing, School of Health Sciences, Cyprus University of Technology, Limassol, Cyprus; 3 School of Translational Medicine, University of Manchester, Manchester, UK; 4 Department of Pediatrics, Areteion Hospital, Nicosia, Cyprus; 5 Third Department of Pediatrics, University of Athens School of Medicine, Attikon Hospital, Athens, Greece; 6 Department of Environmental Health, Harvard School of Public Health, Boston, Massachusetts, USA; 7 Maryland Institute for Applied Environmental Health, University of Maryland, College Park, Maryland, USA

## Abstract

**Background:**

The prevalence of childhood asthma and allergies in Cyprus was significantly higher in urban compared to rural areas back in the year 2000, against a background of an overall low prevalence (e.g. current wheeze 6.9%) by comparison to northern European countries. In this study we aimed to assess temporal changes in the prevalence of asthma and allergies in Cyprus after an 8-year interval and to examine whether any differential changes have occurred in urban and rural parts of the island.

**Methods:**

During the academic years 1999-2000 and 2007-2008, the parents of 7-8 year old children residing in the same set of urban and rural areas completed the ISAAC core questionnaire. In addition to providing prevalence estimates of allergic diseases in 2000 and 2008, changes between the two periods were expressed as odds ratios estimated in multiple logistic regression models adjusting for survey participants' characteristics.

**Results:**

The prevalence of current wheeze was higher in 2008 (8.7%, 95% confidence interval 7.5%-9.9%, n = 2216) than the previously recorded figure in 2000 (6.9%, 95% CI 6.2%-7.6%, OR = 1.25, 95% CI: 1.02-1.53, n = 4944). Significant increases were also seen in the prevalence of lifetime asthma (11.3% vs. 17.4%, OR = 1.59, CI: 1.36-1.86), eczema (6.8% vs. 13.5%, OR = 1.91, CI: 1.59-2.29) and allergic rhinoconjuctivitis (2.6% vs. 5.2%, OR = 1.82, CI: 1.39-2.41). The prevalence of current wheeze nearly doubled between 2000 and 2008 in rural areas (5.4% vs. 9.7%, OR 1.81, CI: 1.24-2.64) while no significant change was observed in urban areas (7.5% vs. 8.4%, OR 1.08, CI: 0.84-1.37); p value for effect modification = 0.04. Rises in asthma and rhinitis prevalence, but not eczema were also more pronounced in rural compared to urban areas.

**Conclusions:**

The prevalence of allergic diseases in Cyprus is still on the rise; recent increases appear more pronounced among children living in rural areas possibly indicating recent environmental and lifestyle changes in these communities

## Background

The epidemiology of asthma and allergies has been investigated in many parts of the world by the International Study of Asthma and Allergies in Childhood (ISAAC). ISAAC Phase I in the early 1990s has shown wide between-country variations in the prevalence of asthma and allergies, even amongst populations of similar genetic background [1,2] whilst ISAAC Phase III provided valuable information on temporal changes of allergic disease prevalence 5-10 years later [3]. The observed patterns were attributed to environmental exposures and lifestyle changes [4,5] of genetically susceptible individuals including exposure to urban and rural residence. Indeed a number of studies have provided evidence to suggest that the prevalence of asthma, allergies and atopic sensitization is lower in rural areas [6,7]. This was mostly attributed to the "farming effect" [8-12], although not all farming environments were shown to be protective [13]. On the other hand, a number of factors relating to the urban environment were shown to increase the risk of asthma and allergies [6,14,15], such as exposure to air pollution [16], a more hygienic environment in early life (i.e. fewer infections) [17], diet [18] and obesity [19]. Furthermore, in a recent study from Greece, the "urban effect" was implicated in the higher atopic sensitization observed in urban areas even in comparison to rural non-farming areas [14]. Therefore, it is still not clear which particular aspects of an urban Vs a rural environment affect the development of allergies. Numerous studies have assessed time trends in the prevalence of asthma and allergies worldwide, yet few have investigated such changes differentially in urban and rural areas within the same population to assist our understanding of the role of such environments in disease development.

In the Republic of Cyprus, the first ever investigation in year 2000 of the prevalence of asthma and allergies in children aged 7-8 years using the ISAAC questionnaire showed an overall low prevalence of current wheeze at 6.9%, consistent with findings from countries within the region like Greece [20], Turkey [21], Italy and Malta [5]. The prevalence of allergies was shown to be significantly higher in the urban compared to the rural areas [22] despite the small size of the island and largely genetically homogenous population in the areas controlled by the Republic of Cyprus. Against a background of declining rates of agricultural and farming practices in rural areas of the island [23] and a more uniform uptake of a more westernized way of living in recent years, we aimed (a) to assess overall temporal changes in the prevalence of asthma and allergies in Cyprus and (b) to investigate whether changes in the prevalence of allergies have been differential in the urban and rural parts of the country.

## Methods

### Study population and design

During the academic years (i.e. October to March) 1999-2000 and 2007-08, we invited all children enrolled in the second grade of public primary schools (i.e. aged 7-8 years) who resided in the two main districts of the Republic of Cyprus, Nicosia and Limassol, to participate in this study. Trained field workers contacted the school Head Teacher and arranged to visit the schools where they distributed the study material to the children. In total, 71 schools (40 urban and 31 rural) in the Nicosia and 56 schools (33 urban and 23 rural) in the Limassol district were contacted by the field workers. All but 3 of these schools agreed to participate. The study questionnaire was completed by the children's parents and was returned to school for collection by the field worker, together with a signed consent form as requested by the Cyprus National Bioethics Committee who approved the study.

### Study questionnaire

In both studies, we used the Greek translation of the International Study of Asthma and Allergy in Childhood (ISAAC) core questionnaire [24]. The ISAAC questionnaire, a widely used tool in large scale studies for the purpose of assessing asthma and allergy symptoms, enabled us to make comparisons - both internal (since this was also the tool used in the original 2000 study) as well as with other countries within and beyond the Eastern Mediterranean region. The questionnaire was supplemented with questions on socio-demographic and risk characteristics such as the country of birth (Cyprus or abroad), exposure to parental smoking at home (Is either the mother or father of the child smoking at home at this point in time?), exposure to maternal smoking during pregnancy (Has the mother been smoking during the pregnancy of this child?) and family history of atopy (Has any member of the immediate family - father, mother, sibling- ever had asthma, eczema or allergic rhinitis?).

We also collected information on the residential address of the study participants at the time of the survey. The place of residence was characterized as urban if the family address was (a) within the boundaries of the metropolitan areas of Nicosia or Limassol (the two largest cities on the island) or (b) in any of the municipalities in suburban areas that are in geographic continuity with the inner city. In this way nine municipalities were classified as urban in the district of Nicosia with an average population size of 23300 inhabitants as recorded in the last population census (range 2492-58520) and mean population density of 1770 people per km^2 ^(range 233-5809). In Limassol, there were six such municipalities with an average population of 24620 inhabitants (range 3741-94250) and mean population density 1954 people per km^2 ^(range 679-4363). If the address of the family was outside the main cities or their suburbs, the place of residence was characterized as rural. With an extensive network of rural elementary schools, it is very rare for a child in this age-group to reside in rural area but attend an urban school. The catchment area of the 54 rural schools includes a total of 96 rural communities in the Nicosia district and 106 in Limassol. The average number of inhabitants across rural communities in Nicosia is 760 (range 3-5834) and in Limassol 470 (range 2-6435) whilst the mean population density is 63 people per km^2 ^(range 0.5-332) in Nicosia and 54 (range 0.4-891) in Limassol [25].

### Definition of study outcomes

We looked at the following asthma and allergy outcomes as defined below in consistency with previous studies [3]

**Ever Wheeze**: Positive answer to the question "Has your child ever had wheezing?"

**Current Wheeze**: Positive answer to the question "Has your child had wheezing in the last 12 months?"

**Asthma diagnosis**: Positive answer to the question "Has your child ever had asthma?"

**Severity of asthma: **Positive response to the questions referring to the following: ≥ 4 attacks of wheeze in last 12 months and/or ≥ 1 night per week sleep disturbance and/or wheeze affecting speech in the last 12 months

**Current eczema**: Positive answers to both questions: "Has your child had this itchy rash at any time in the last 12 months" and "Has this itchy rash at any time affected any of the following places: the folds of the elbows, behind the knees, in front of the ankles, under the buttocks or around the neck, ears or eyes"

**Diagnosis of eczema**: Positive answer to the question "Has your child ever had eczema"

**Current Allergic Rhinoconjuctivitis**: Positive answer to both questions "In the past 12 months, has your child had a problem with sneezing or a runny or a blocked nose when he/she did not have a cold or the flu?" And "In the past 12 months, has this nose problem been accompanied by itchy watery eyes"

**Diagnosis Hay Fever: **Positive answer to the question "Has your child ever had allergic rhinitis?"

### Statistical analysis

Differences in the socio-demographic composition of the two study samples (overall and stratified by urban/rural residence) were investigated using chi-squared tests. As per ISAAC protocol, we calculated prevalence point estimates of all study outcomes (overall as well as urban/rural-specific) by dividing positive responses to the given question by the total number of questionnaires (missing and inconsistent responses were included in the denominator for the prevalence calculations) and expressed them as percentages; 95% confidence intervals around these estimates were calculated using the normal large-sample approximation for the standard error of a single proportion.

Furthermore, we assessed temporal changes in the overall prevalence of study outcomes in the eight year period. As a measure of the magnitude of the change in study outcomes between the two surveys, we calculated crude and adjusted odds ratios (and 95% CI) of an outcome in the 2008 as compared to the 2000 survey in multivariable logistic regression models. This enabled us to adjust the estimates for possible confounding due to differences in study populations by important socio-demographic characteristics, which may also be a product of sampling differences between the two surveys. We controlled for a priori variables known to be associated with outcomes and exposures of interest (i.e. year of survey and urban/rural samples) such as sex, family history of atopy, history of parental smoking at home, maternal smoking in pregnancy, country of birth, district residence and urban/rural residence where appropriate. We extended models to include an interaction term between survey year and place of residence in order to assess any evidence of modification in the magnitude of the observed temporal effect in urban Vs rural areas. Finally, due to the lower participation rate in the 2008 survey we performed post-hoc sensitivity analyses to quantify the potential effect of selection bias on the observed estimates. Specifically, we adopted a probabilistic approach in order to adjust our OR estimates for the observed urban or rural-specific temporal changes between 2000 and 2008 assuming a prior distribution for the selection bias factor with 95% probability of the bias being in the range of 0.6 to 1.7. Only to give an example, a selection bias factor of 1.7 refers to a 1.7-fold higher likelihood of selecting a "case" rather than a "non-case" in 2008 compared to 2000. If, say, there was an equal likelihood of selecting current wheezers and non-current wheezers (or any other outcome for that matter) in the 2000 survey (perhaps, around the overall 82% observed), this would correspond to more than 90% probability of selecting current wheezers in 2008 but only about half among non-current wheezers. Furthermore, we used a deterministic approach in order to pinpoint to the magnitude of the bias factor that would overturn the urban-rural pattern as observed in 2008 (i.e. cross over, below or past, OR = 1) and arrive at the levels observed in 2000 [26]. Sensitivity analyses were performed in STATA (using the *episens *command) while all other statistical analyses of the data was performed using the SPSS 16 software package. Statistical significance was set at p < 0.05.

## Results

### Population characteristics

A total number of 4944 children (82% of those invited) participated in the 2000 survey and 2216 (48% of those invited) in the 2008 survey. We present the demographic and social characteristics of the two study populations in Table 1. The gender distribution was similar between the two surveys as was the percentage of children who were born in Cyprus and whose mother was smoking during pregnancy. However, compared to the 2000 survey, there was a higher participation from the district of Nicosia (rather than Limassol) in year 2008 (66% vs. 52.9% in 2008 and 2000 respectively, p < 0.01) and lower participation from urban as opposed to rural areas (65.8% vs. 73.5%, p < 0.01). Report of family history of atopy appeared slightly more frequent in 2008 compared to year 2000 (37% vs. 34.1%, p < 0.05) whereas exposure to parental smoking at home appeared lower in year 2008 (39.9% vs. 48.0%, p < 0.01).

**Table 1:** Population characteristics in the 2000 and 2008 surveys

	Year 2000 n = 4944 (82% of invited) % (CI)	Year 2008 n = 2216 (48% of invited) % (CI)
Sex (female)	50.3 (48.9-51.7)	49.4 (47.3-51.5)
Residence (urban)	73.5 (72.3-74.7)	65.8** (63.8-67.8)
District Residence (Nicosia)	52.9 (51.5-54.3)	66.0** (64.0-68.0)
Country of Birth (Cyprus)	94.9 (94.3-95.5)	95.3 (94.4-96.2)
Positive Family History of Atopy	34.1 (32.8-35.4)	37.0* (35.0-39.0)
Parental smoking at home	48.0 (46.6-49.4)	39.9** (37.9-41.9)
Maternal Smoking in Pregnancy	2.3 (1.9-2.7)	2.9 (2.2-3.6)

*, ** p < 0.05 and p < 0.01 respectively in chi-square tests comparing differences between participant characteristics between years 2000 and 2008

In Table 2, we present the socio-demographic characteristics of the study participants stratified by urban and rural residence in each survey. In both study populations, the similarities as well as differences in the characteristics of the children residing in urban and rural areas appear consistent. More specifically, in either survey, sex distribution and district residence were similar between children living in urban and rural areas. Children living in rural areas had lower report of family history of atopy (p < 0.01) and were more likely to have been born in Cyprus (p < 0.01) in both population samples. We also observed smaller but consistent differences between urban and rural areas in both surveys in other covariates such as the exposure to parental smoking at home and maternal smoking during pregnancy although these did not reach statistical significance.

**Table 2:** Population characteristics of children living in urban and rural areas in the 2000 and 2008 surveys

	Year 2000		Year 2008	
	**Urban** **n = 3600** **%**	**Rural** **n = 1296** **%**	**Urban** **n = 1410** **%**	**Rural** **n = 732** **%**

Sex (female)	50.6	49.2	50.1	48.4
District Residence (Nicosia)	53.0	52.9	67.4	69.3
Country of Birth (Cyprus)	94.6	97.1**	94.1	97.5††
Family History of atopy	35.2	31.0**	38.4	30.7††
Parental Smoking at home	47.9	48.7	38.4	42.5
Maternal smoking in pregnancy	2.6	1.5*	3.3	2.0

*, ** p < 0.05 and p < 0.01 respectively of chi-square test comparing participants characteristics among those living in urban and rural areas at the 2000 survey

†, †† p <0.05 and p < 0.01 respectively of chi-square test comparing participant characteristics among those living in urban and rural areas at the 2008 survey

### Temporal changes in prevalence between years 2000 and 2008

In Table 3, we present the prevalence of asthma and allergy symptoms or lifetime disease diagnostic labeling in years 2000 and 2008 as well as unadjusted and adjusted odds ratios of the magnitude of the change in study outcomes between the two surveys. We found a significant increase in the prevalence of ever and current wheezing, ever asthma as well as severity of asthma between the two survey years as projected by the crude OR estimates. Furthermore, the estimates remain largely unaffected even after adjusting for the socio-demographic characteristics of the participants in the two surveys. In particular, the prevalence of current wheeze appears to have risen from 6.9% to 8.7% (adjusted OR 1.25, CI 1.02-1.53, p = 0.03) and ever wheeze from 18.5% to 25% (adjusted OR 1.41, CI 1.23-1.61, p < 0.01); this suggests a significant increase in the odds of reporting current wheeze and ever wheeze by 2008 compared to 2000 in the order of 25% and 41% respectively. The prevalence of lifetime asthma also appeared to have increased from 11.3% to 17.4% (adjusted OR 1.59, CI 1.36-1.86, p < 0.01) and severity of asthma from 1.5% to 2.6% (adjusted OR 1.75, CI 1.19-2.55, p < 0.01). Similarly, the prevalence of current symptoms of eczema and eczema ever also increased between the two time points; current eczema prevalence showed a small but significant rise from 3.7% to 4.6% (OR 1.36, CI 1.04-1.78, p = 0.03). Eczema ever almost doubled from 6.8% in year 2000 to 13.5% in 2008 (OR 1.91, CI 1.59-2.29, p < 0.001) i.e. in year 2008 parents were almost twice more likely to report a diagnosis of their child ever having eczema compared to year 2000 even after adjusting for compositional differences in the study samples between the two study years. Allergic rhinitis ever also doubled from 2.6% to 5.2% (OR 1.82, CI 1.39-2.41, p < 0.001), whilst the prevalence of current symptoms of rhinoconjuctivitis displayed only a small and non significant increase from 2.1% in 2000 to 2.5% in 2008 (OR 1.11, CI 0.78-1.58, p = 0.57). Overall, other than current symptoms of rhinoconjuctivitis, from the smallest (but statistically significant) increase of 25% in the case of current wheeze to almost double in the case of eczema diagnosis ever, we observed consistent rises in all study outcomes between the two surveys. There was no statistical evidence that the magnitude of effect (i.e. change between study periods) was different in children with and without family history of allergies for any of the study outcomes - not presented in detail.

**Table 3:** Prevalence of asthmatic and allergic symptoms and diagnostic labeling in the 2000 and 2008 surveys

	Year 2000	Year 2008	Year 2000 vs 2008 Unadjusted		Year 2000 vs 2008 Adjusted*	
	% (95% CI)	% (95% CI)	OR (CI)	P-Value	OR (CI)	P-Value
Ever Wheeze	18.5 (17.4-19.6)	25 (23.2-26.8)	1.42 (1.26-1.61)	P <0.01	1.41 (1.23-1.61)	P <0.01
Current Wheeze	6.9 (6.2-7.6)	8.7 (7.5-9.9)	1.25 (1.04-1.51)	P = 0.02	1.25 (1.02-1.53)	P = 0.03
Diagnosis Asthma	11.3 (10.4-12.2)	17.4 (15.8-19.0)	1.62 (1.40-1.86)	P <0.01	1.59 (1.36-1.86)	P <0.01
Severity of Asthma	1.5 (1.2-1.8)	2.6 (1.9-3.3)	1.79 (1.27-2.54)	P < 0.01	1.75 (1.19-2.55)	P < 0.01
Current Eczema	3.7 (3.2-4.2)	4.6 (3.7-5.5)	1.28 (0.99-1.64)	P = 0.05	1.36 (1.04-1.78)	P = 0.03
Diagnosis Eczema	6.8 (6.1-7.5)	13.5 (12.1-14.9)	2.11 (1.79-2.48)	P <0.01	1.91 (1.59-2.29)	P <0.01
Current Allergic Rhinoconjuctivitis	2.1 (1.7-2.5)	2.5 (1.8-3.2)	1.20 (0.86-1.66)	P = 0.29	1.11 (0.78-1.58)	P = 0.57
Diagnosis of Rhinitis	2.6 (2.1-3.1)	5.2 (4.3-6.1)	1.98 (1.54-2.56)	P <0.01	1.82 (1.39-2.41)	P <0.01

* Adjusted for sex, urban/rural residence, district residence, family history of atopy, history of parental smoking at home, maternal smoking in pregnancy and country of birth

### Temporal changes in prevalence by urban and rural residence

**Figure 1:**
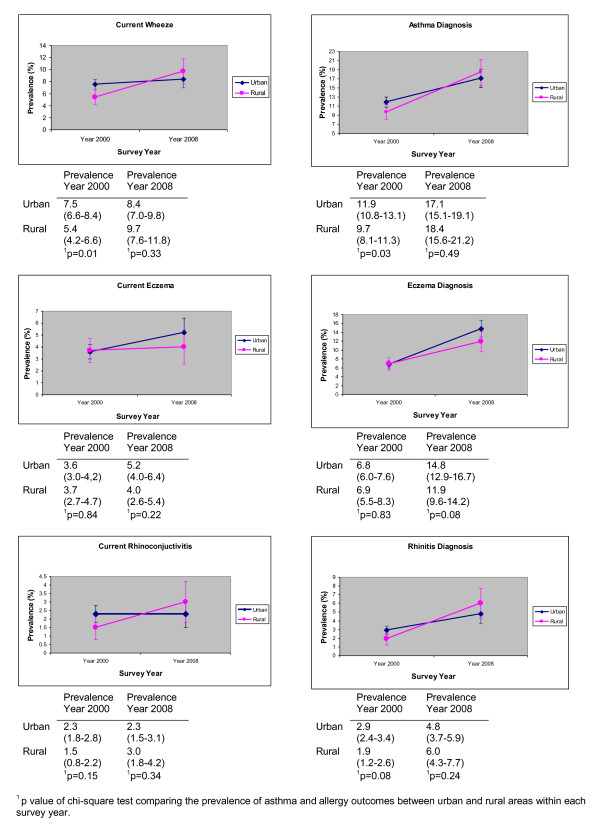
**Urban-rural differences in the prevalence of asthma and allergies in 2000 and 2008 surveys**.

In Figure 1, the observed changes in the prevalence of asthma and allergies between years 2000 and 2008 in urban and rural parts of the island separately are presented graphically. Generally, the prevalence of most asthma and allergy outcomes appeared increased in both urban and rural areas by the year 2008 compared to year 2000. However, in the case of asthma and allergic rhinitis outcomes rises appear more pronounced in the rural than the urban areas while the opposite is true for eczema outcomes. As a result, in the case of asthma and allergic rhinitis outcomes differences between urban and rural areas have become smaller in 2008 compared to year 2000 whereas in the case of current symptoms of eczema and eczema ever prevalence differences appear wider.

Unadjusted and adjusted estimates for the magnitude of the urban Vs rural-specific 2000-to-2008 change in the odds of reporting asthma and allergy outcomes (see Table 4) confirm that the change was larger in rural compared to urban areas at least for asthma and rhinitis symptoms or lifetime diagnosis. For instance, the odds of reporting current wheezing among children in rural areas have almost doubled since the 2000 survey (adjusted OR 1.81, CI: 1.24-2.64) whereas no significant increase was observed in the urban areas (OR 1.08, CI: 0.86-1.46); p-value for effect modification = 0.04. Rises also appeared more pronounced in the rural than the urban areas for asthma ever (p value for effect modification = 0.02), severity of asthma (p value for effect modification = 0.05) and current allergic rhinoconjuctivitis (p value for effect modification = 0.02) whilst there is some weaker evidence that the increase in allergic rhinitis ever prevalence was also larger in rural areas compared to urban areas (p value for effect modification = 0.07). In contrast, higher rises were seen in the prevalence of eczema outcomes in the urban compared to the rural areas between the two survey years although these were not significantly different either in the case of current eczema (p value for effect modification = 0.62) or eczema ever (p value for effect modification = 0.14).

**Table 4:** Differences in the temporal changes of asthma and allergies prevalence in urban and rural areas between years 2000 and 2008

	Year 2000 vs Year 2008				
	**URBAN RESIDENCE**		**RURAL RESIDENCE**		
	**OR** **(CI)** **P-Value** **Unadjusted**	**OR** **(CI)** **P-Value** Adjusted*	**OR** **(CI)** **P-Value** **Unadjusted**	**OR** **(CI)** **P-Value** Adjusted*	**Effect modification§**

**Ever Wheeze**	1.40 (1.21-1.62) P <0.01	1.33 (1.13-1.55) P <0.01	1.55 (1.25-1.94) P <0.01	1.66 (1.30-2.13) P <0.01	P = 0.35
**Current Wheeze**	1.11 (0.89-1.39) P = 0.37	1.08 (0.84-1.37) P = 0.56	1.84 (1.30-2.59) P <0.01	1.81 (1.24-2.64) P <0.01	P = 0.04
**Diagnosis Asthma** **Ever**	1.50 (1.26-1.78) P <0.01	1.39 (1.15-1.68) P <0.01	2.04 (1.57-2.65) P <0.01	2.20 (1.64-2.94) P <0.01	P = 0.02
**Severity of Asthma**	1.41 (0.89-2.22) P = 0.14	1.30 (0.80-2.11) P = 0.29	2.67 (1.48-4.82) p < 0.01	2.99 (1.54-5.58) p < 0.01	P = 0.05
**Current Eczema**	1.45 (1.08-1.95) P = 0.01	1.46 (1.07-2.00) P = 0.02	1.06 (0.66-1.70) P = 0.80	1.14 (0.68-1.89) P = 0.62	P = 0.32
**Diagnosis Eczema** **Ever**	2.33 (1.91-2.84) P <0.01	2.05 (1.66-2.54) P <0.01	1.79 (1.31-2.44) P <0.01	1.58 (1.12-2.22) P = 0.01	P = 0.14
**Current Allergic** **Rhinoconjuctivitis**	1.01 (0.67-1.52) P = 0.96	0.84 (0.54-1.29) P = 0.42	1.93 (1.05-3.57) P = 0.04	2.25 (1.16-4.37) P = 0.02	P = 0.04
**Diagnosis Rhinitis** **Ever**	1.66 (1.21-2.26) P <0.01	1.54 (1.10-2.14) P = 0.01	3.10 (1.88-5.11) P <0.01	2.79 (1.64-4.75) P <0.01	P = 0.07

* Adjusted for sex, country of birth, district residence, family history of atopy, parental smoking at home, maternal smoking in pregnancy

§ Effect modification of the temporal changes in asthma and allergy outcomes in years 2000 and 2008 by urban and rural residence adjusting for sex, country of birth, district residence, family history of atopy, parental smoking at home and maternal smoking in pregnancy

Probabilistic sensitivity analyses provided further support that the changes observed in rural areas between 2000 and 2008 are relatively robust to possible differential selection bias, at least with a selection bias factor in the range 0.6 to 1.7. With the exception of current rhinitis and current eczema (for which no change between the 2000 Vs 2008 surveys was observed in rural areas), for all other outcomes the 95% simulation limits of the systematic and random error-adjusted OR for the rural-specific 2000 Vs 2008 change excluded the value 1. For example, in the case of current wheeze, the 95% limits for the bias-adjusted OR for the observed change in rural areas was (1.01, 3.46), with the lower limit reflecting the scenario described above. Diagnoses outcomes were even more robust to higher degrees of bias. Furthermore, focusing on the urban-rural differences as observed in 2008, sensitivity analyses have indicated that a bias factor lower than 0.8 would have made the anyway non-significant OR of current wheeze of 0.84 (i.e. 8.4% Vs 9.7) in favour of urban areas cross over past an OR = 1 while a factor lower than 0.6 would result to a masking of a higher prevalence in urban areas of the magnitude observed in 2000 (i.e. 7.5% Vs 5.4%). Assuming, say, that the probability of non current-wheezers participating in the survey was close to the overall 2008 response rate of 45% and 60% observed in urban and rural areas respectively, a bias factor of this magnitude would translate to a difference in the probability of selecting current wheezers of 40% in urban areas (i.e. lower than the overall response rate) Vs 90% in rural areas (i.e. nearly everyone).

## Discussion

### Main Findings

In this study, we investigated temporal changes in the prevalence of asthma and allergies in the two main districts of the Republic of Cyprus based on two cross-sectional surveys eight years apart. We have also assessed whether any changes that occurred in this period were differential between the urban and rural areas of the island. With the exception of current symptoms of rhinoconjuctivitis, the prevalence of all outcomes investigated appeared significantly increased in 2008 compared to 2000. In fact, the prevalence of lifetime diagnosis of asthma, eczema and rhinitis have almost doubled. Rises were generally observed in both urban and rural areas but, with the exception of eczema symptoms and diagnosis, these consistently appeared more pronounced in the rural areas of the country.

### Limitations

In contrast to the high participation rate observed in the first survey, participation was quite low in the subsequent 2008 survey. Anecdotal evidence among research teams in Cyprus points to an increased reluctance in recent years to participate in research studies resulting in lower response rates compared to a decade ago. It is likely that a combination of factors are responsible for this including (a) an increasing reluctance of parents to offer personal information and (b) a recent steady rise in the number of surveys among the school population, which in a country the size of Cyprus may be important.

We observed a lower participation rate in urban as opposed to rural areas (44.5% Vs 59.5% respectively). In the absence of information on non-responders, or any data from official sources more recent than the 2001 census, for the purpose of contrasting the socio-demographic profile of the study participants, we identified a 2004 study on childhood obesity among Cypriot children aged 2-6,. The time frame and age band of the participants that would have participated four years later at age 8 in our 2008 survey were captured by that study with a participation rate of over 75% [27]. Parental level of education as reported in that study (as well as several other participant characteristics such as birth weight) compares favourably with the figures observed in our study sample. This is the case both in terms of the overall sample as well as when broken down to urban-rural areas with the same approach as in our study. This serves as an indication that the low response rate in 2008 did not seem to compromise the representativeness of our study population. Although it is likely that affected children may have an incentive to participate in the survey, we have no reason to believe that this should be differential in urban Vs rural areas (i.e. there is no reason why proportionally more asthmatics than non-asthmatics from the urban areas would have participated in the study but the opposite to have been the case in rural areas). In any case, sensitivity analyses have indicated that, even though not impossible, such differential participation would have to be quite marked for the observed rise in rural areas across several study outcomes to be an artifact.To some extent, this is also reinforced by the fact that the two samples appear internally consistent both in terms of stable characteristics (e.g. the proportion of children born in Cyprus is significantly higher in the rural compared to the urban areas in both surveys) as well as observed changes between the two survey years (e.g. a decrease in parental smoking in the home occurred in both urban and rural areas). Finally, in 2001 (when the participation rate was over 80%) the observed difference between urban and rural areas was clearly striking. This was not the case by the year 2008, both before and after adjusting for differences in the composition of our two samples, serving at least as an indication that the previously wide urban-rural gap may have now narrowed.

Limited by its cross-sectional design, no inferences can be made on the causal association of risk factors assessed in the two surveys with the study outcomes. For instance, the observed increase in the report of family history of atopy between the two surveys may represent a "real" change, as has been seen in other studies [21], or may simply be the result of sampling. However, we adjusted for family history of atopy in our assessment of changes in prevalence of asthma and allergy outcomes among the children so that this potential source of error has been accounted for. While it was important to adjust for the possible confounding effect due to compositional differences in the study populations, we have made no attempt to associate changes in any environmental or lifestyle factors with the increase in the asthma and allergy outcomes in the urban and rural areas of Cyprus. Of course, residual confounding by other important factors, such as socioeconomic status or exposure to animals and/or farming practices, could not be corrected for in the analysis due to lack of comparable data from the two surveys. Nevertheless, as also mentioned above, while adjustments are necessary to correct for sample differences, it was not the purpose of the study to identify the specific factors related to rural living, or even characteristics of rural populations, which underline these changes. Lastly, we should note that the Greek version of the ISAAC questionnaire has not been officially validated. However, there is only one accepted version which has been in use as a standard tool ever since in several studies in Greece [24] and asthma reporting using this questionnaire has been found to correlate well with objective measures [28].

### Temporal changes in asthma and allergy outcomes between years 2000 and 2008

This is the first study to have assessed temporal changes in the prevalence of asthma and allergies in Cyprus. We have found that the prevalence of current wheezing symptoms has significantly increased by 0.23% annually to 8.7% in eight years. While still at a level much lower than the global average of 11.6%, this translates to an annual increase greater than the global average of 0.13% [29]. The rise in the prevalence of current wheeze is consistent with findings from studies conducted using the ISAAC questionnaire in neighboring countries such as Greece [20], Turkey [21], Malta [30] and other Eastern European and Mediterranean countries [29]. The annual increase of 0.23% however is lower than the regional average (Eastern Mediterranean) of 0.79% as was shown by the ISAAC Phase III study group which nonetheless only included centers from three countries in this region. In addition, the prevalence of current eczema symptoms increased significantly between the two study periods (from 3.7% to 4.6%); consistent with ISAAC Phase III studies in the same age group which have shown increases in current eczema symptoms in most countries [5]. Current eczema prevalence in Cyprus is close to the Eastern Mediterranean average of 4.8% [31]. The prevalence of current symptoms of allergic rhinitis in Cyprus is very low but comparable nonetheless with Greece and Eastern European countries [32].

The significant increase in the prevalence of lifetime asthma, eczema and allergic rhinitis in Cyprus in eight years is consistent with the rise in prevalence of these outcomes in most parts of the world, even in countries with declining prevalence in current symptoms [29]; to some extent, this can be attributed to improved awareness and diagnostic labeling of these conditions. However, considering the fact that the prevalence of allergic symptoms has also increased considerably in Cyprus, changes between the two time periods may at least partly reflect a true increase. Furthermore, the current prevalence in Cyprus of ever asthma diagnosis (17.4%) and ever eczema (13.5%) is much higher than respective regional averages of 9.1% and 7.2% [5]. In contrast, allergic rhinitis diagnosis prevalence at 5.2% is much lower than the regional average of 13.9% [32]; this may represent lower awareness of this disease in Cyprus or a true low prevalence given the similarly low prevalence of current allergic rhinoconjuctivitis symptoms.

### Temporal changes in asthma and allergy outcomes by urban and rural residence

We have found that the increase in symptoms of asthma and allergic rhinitis in this period may have been specific to rural areas (with the previous urban-rural differences narrowing) whereas current eczema symptoms appeared to have increased significantly in urban areas only. In this study, only the place of residence at the time of the survey was recorded; hence this may not necessarily represent a child's early life exposure, which is important in the development of allergic diseases. However, the purpose of this study was not to investigate whether "exposure" to a rural environment (and which particular aspects of it) poses an independent effect on the development of the study outcomes but to describe the current urban-rural patterns of allergic diseases. In any case, it is unlikely that rises in rural areas are a product of a recent movement of population out of metropolitan areas. At 0.8%, population mobility in Cyprus (defined as the proportion of people living in a different address a year before the census) is particularly low [25].

Studies from urban areas elsewhere in the Eastern Mediterranean region such as the city of Patras Greece [20] and Istanbul, Turkey [33], have indicated continuing rises in current symptoms of all allergic diseases; nevertheless both of these studies were conducted in urban areas during an earlier time period (i.e. between the early 1990s to early 2000), and possibly in populations at an earlier stage of urbanization both at baseline as well as follow-ups, and were perhaps still in the process of adopting lifestyle changes that play a role in allergic diseases development.

Only a small number of studies have investigated trends in the prevalence of allergic conditions within the same country population in urban and rural areas separately [10,21,34]. A Swedish study over an earlier period of three decades (1952-1981) has shown a continuing rise in asthma, eczema and rhinitis diagnosis prevalence among conscripts in both rural and urban areas [10]. Another study from Italy, investigating asthma and allergy trends between 1994-2002 has found the increase in the prevalence of allergic rhinitis and eczema symptoms to be higher in adolescents living in large metropolitan areas compared to areas with lower population density [34]. Furthermore, a more recent study from Edirne Turkey which investigated prevalence of allergies differentially in urban and rural areas between 1994-2004, showed the prevalence of current wheeze to have increased significantly in both urban and rural areas [21]. In contrast to our findings, the Edirne study showed the rise in the asthma and allergic rhinitis outcomes to be greater in the urban rather than the rural areas. Unlike the Edirne study, rural areas in our study are not defined in terms of farming practices (but in terms of their population size and remoteness from big centres of population) and include places in transition from a more rural to a more urban/westernized way of living.

In Sweden [10] and in several earlier studies [8,9], living on a farm has been shown to be associated with reduced risk of symptoms relating to upper and lower airways but not eczema. Therefore, it wouldn't be unreasonable to assume that as a result of declining rates of farming practice across rural areas of Cyprus, any protective effect of farming on asthma and rhinitis has weakened, explaining the increase in the prevalence of symptoms relating to these diseases but not eczema. Of course, other environmental and lifestyle factors are likely to be implicated in the aetiology of the observed changes. For example, the role of socio-economic factors (and any underlying changes in the socio-economic status of the population in this period) is crucial. Since no socio-economic data were recorded in the 2000 survey, we could not assess whether any significant changes in the socio-economic profile of the population occurred during this period. According to the Cyprus Statistical Service survey on Income and Living Conditions, population socio-economic indicators (income, poverty, education, etc) have been very stable between 2005-2008 [35]. In any case, not adjusting for any potential differences in the socio-economic status of the children might have introduced uncontrolled confounding. We used data on parental level of education (available from the 2008 survey) in order to assess the extent to which socio-economic differences between participants in urban and rural areas explain the observed 2008 urban-rural patterns. Despite the fact that, as expected, educational attainment was higher in urban areas (i.e. 59.6% Vs 40.3% of participants with one parent with tertiary education respectively), this did not seem to affect our findings, indicating that the observed diversion (e.g. in the case of eczema) or narrowing (e.g. in the case of wheezing) in 2008 is not accounted by socio-economic differences between urban and rural parts of the country.

Generally, in the absence of detailed information on lifestyle and environmental factors which may be implicated in the differential epidemiology of allergic diseases in Cypriot children, we can only speculate that the higher increase in allergies in the rural areas may be the result of a general trend of reduced exposure to farming and/or, in parallel, an increased adoption of a more "urban" lifestyle among families in rural areas related to diet patterns, reduced exercise levels and a more hygienic environment.

## Conclusions

The symptoms and disease labeling prevalence of asthma and allergies in Cyprus is still on the rise. In this descriptive study we investigated whether temporal changes in the prevalence of allergic diseases were differential between populations residing in urban and rural areas. By 2008, the risk of asthma and rhinitis but not eczema appeared to have been increasing more among children living in the rural than the urban areas of the island. Further studies are needed to investigate the environmental and lifestyle factors implicated in the changes in asthma and allergy prevalence in Cyprus as well as monitoring future trends.

## Competing interests

The authors declare that they have no competing interests.

## Authors' contributions

OK supervised data collection in the 2008 study, performed the statistical analysis and prepared the first draft of the manuscript. PY conceived, designed and supervised the conduction of both studies. PY and NM assisted in drafting and revising the manuscript. NM advised with the statistical analysis. DKM and SP took part in the design phase of the 2008 and 2000 study respectively. NN supervised data collection during the 2000 study. KNP and DKM critically revised the manuscript. All authors have read and approved the final version of the manuscript.

## Pre-publication history

The pre-publication history for this paper can be accessed here:

http://www.biomedcentral.com/1471-2458/11/858/prepub
